# Allometry and morphological trait relationship in the sexually dimorphic Chinese dobsonfly, *Acanthacorydalisasiatica* (Wood-Mason, 1884) (Megaloptera, Corydalidae)

**DOI:** 10.3897/zookeys.854.32897

**Published:** 2019-06-10

**Authors:** Chengquan Cao, Pei Yu, Fumio Hayashi

**Affiliations:** 1 College of Life Science, Leshan Normal University, Leshan, Sichuan 614004, China; 2 Department of Biology, Tokyo Metropolitan University, Minamiosawa 1-1, Hachioji, Tokyo 192-0397, Japan

**Keywords:** Male genitalia, morphological trade-off, sexually selected trait, static allometry

## Abstract

Male insects with large weapons such as horns and elongate mandibles would be expected to invest more on such structures than other parts of the body for advantages in male to male competition for mating. In male genitalia, however, intermediate size provides a better fit for more females than small or large sizes, and such a male would leave more offspring regardless of their body size. These predictions were tested using a static allometry analysis between body size and other trait sizes. *Acanthacorydalisasiatica* is a large dobsonfly (Megalotera) and males have conspicuously large mandibles used as weapons. We examined the hypothesis that the male mandibles of this sexually dimorphic species are sexually selected to enlarge, whereas the male genitalia are stable to be intermediate regardless of a great variation in body size. The results, as predicted, showed positive allometry between male body size and mandible length but negative allometry between male body size and ectoproct length (a male grasping structure). Sperm are transferred through a small spermatophore attached externally to the female genital opening, so it may be evolutionarily unnecessary to develop an enlarged male genital size. In contrast, there may be a trade-off between male mandible size and wing length, because of negative allometry between body size and wing length in males but isometry between them in females.

## Introduction

Many groups of animals develop secondary sexual traits mostly in males but a few in females (reviewed by Emlen 2008; Rico-Guevara and Hurme 2019). In insects, exaggeration in male mandibles, horns, legs, and eye spans is well known, and sexual selection is recognized as a key driver of it (Emlen and Nijhout 2000; Lavine et al. 2015). Studies of the morphological evolution of sexually selected traits often attempt to explain how selection has shaped them, and static allometry has been a useful tool in generating hypotheses about selection on morphology (Eberhard et al. 2009, 2018). Static allometry is a measure of the proportional size of a particular structure in a population of conspecific individuals that have different body sizes but are at the same ontogenetic stage (Eberhard et al. 2009, 2018; Voje 2016; O’Brien et al. 2018). The allometric equation is represented as *y* = a*x*^b^, where a and b are constants (Huxley 1932). In the log-log relationship between body size (*x*) and one body trait (*y*) (log_10_*y* = blog_10_*x* + log_10_ a) in conspecific individuals, the three kinds of relationships arise depending on the slope b of this regression; positive allometry (b > 1) in which larger individuals show disproportionately large traits, negative allometry (b < 1) in which larger individuals show disproportionately smaller traits, and isometry (b = 1) in which the trait size increases proportionately with body size. There are usually isometric relationships between body size and other body parts. In this case, the body proportion does not differ between small and large individuals. Male sexually selected traits such as horns, mandibles, and visual display devices have generally positive allometry with body size (reviewed by Emlen and Nijhout 2000; Kodric-Brown et al. 2006; Bonduriansky 2007; Voje 2016; Eberhard et al. 2018; O’Brien et al. 2018), although the presence or absence of positive allometry cannot be used simply to infer the presence or absence of sexual selection if the function of the traits is unknown (e.g., Bonduriansky 2007). On the other hand, male genital size shows negative allometry or little correlation with male body size in most insect species examined, offering a one-size-fits-all hypothesis of male genital size (reviewed by Eberhard et al. 1998, 2018; Eberhard 2009; Voje 2016). If intermediate-sized genitalia provide a better fit or better tactile stimulation for more females in the population than small genitalia or large genitalia, males with intermediate-sized genitalia would leave more offspring regardless of their body size (Eberhard et al. 1998).

The order Megaloptera is a minor insect group including only two families, 35 genera, and 397 species in the world (Rivera-Gasperín et al. 2019), and little attention has been paid to its behavior. However, this order of insects includes three genera in which the male develops exaggerated traits and conspicuous sexual dimorphism is known. In *Corydalus*, distributed in North to South America, and *Acanthacorydalis*, known from Asia, positive allometry is reported between male body size and mandible size (Liu et al. 2015; Álvarez et al. 2017). These males are known to combat with their mandibles (Liu et al. 2015; Álvarez et al. 2017). In *Platyneuromus* in Central America, the exaggerated male postocular flanges show the positive allometry with male body size, although the function of this postocular flange is still unknown (Liu et al. 2015; Ramírez-Ponce et al. 2017). Thus, sexual selection may affect the development of male mandibles and postocular flanges of these insects. However, the morphometric data sets of previous studies were based on small sample size collected from a variety of localities, because it is usually difficult to collect them in the field. Samples combined multiple populations may mislead the obtained results if local adaptations occur, particularly in the case that some populations are large while others are small in body size. For best understanding of the phenomenon of sexual dimorphism, the morphometric analysis of a single population and comparative studies along the geographic range of a species must be used. Therefore, in this study, we reexamined the allometric relationships of male and female morphological traits of a single population of the Chinese dobsonfly, *Acanthacorydalisasiatica* (Wood-Mason, 1884).

We also examined the allometric relationships of male genital size of this species. In Megaloptera, *Corydalusbidenticulatus* Contreras-Ramos, 1998 is the only species for which the relationship between body and genital size has been studied, and a negative allometry is reported although based on only nine males and statistically marginal at *P* = 0.05 in correlation analysis (Álvarez et al. 2017). Two types of sperm transfer are known in insects; one is ejaculation of sperm or sperm-including spermatophores internally to the female reproductive organs and the other is transfer of sperm via an externally attached spermatophore to the female (Simmons 2001; Chapman 2013). In Megaloptera, males use an external spermatophore (Hayashi 1992, 1993, 1996, 1999; Liu et al. 2015). In this study, the size of male ectoprocts grasping female abdominal tip at mating (Liu et al. 2015) was analyzed allometrically to examine whether this apparatus supports the one-size-fits-all hypothesis (Eberhard et al. 1998). If so, b in the allometric equation is expected <1, and the coefficient of variation (CV) in genital traits should be lower than those in other traits (Eberhard et al. 1998).

## Material and methods

The genus *Acanthacorydalis* includes some of the most remarkable dobsonflies in the world by their large body size and elongated male mandibles (Liu et al. 2005; Cao and Liu 2013). This genus is endemic to Asia and mainly distributed in the Oriental realm, and there are eight species currently recorded from China, India, and Vietnam (Yang and Liu 2010). China has a rich fauna of this genus with six species distributed from southern and southwestern to northern China (Yang and Liu 2010). The larva, an aquatic predator, lives in the relatively large river beds and the final-instar larva leaves water to pupate in the riverside soils (Cao and Liu 2013).

**Figure 1.**
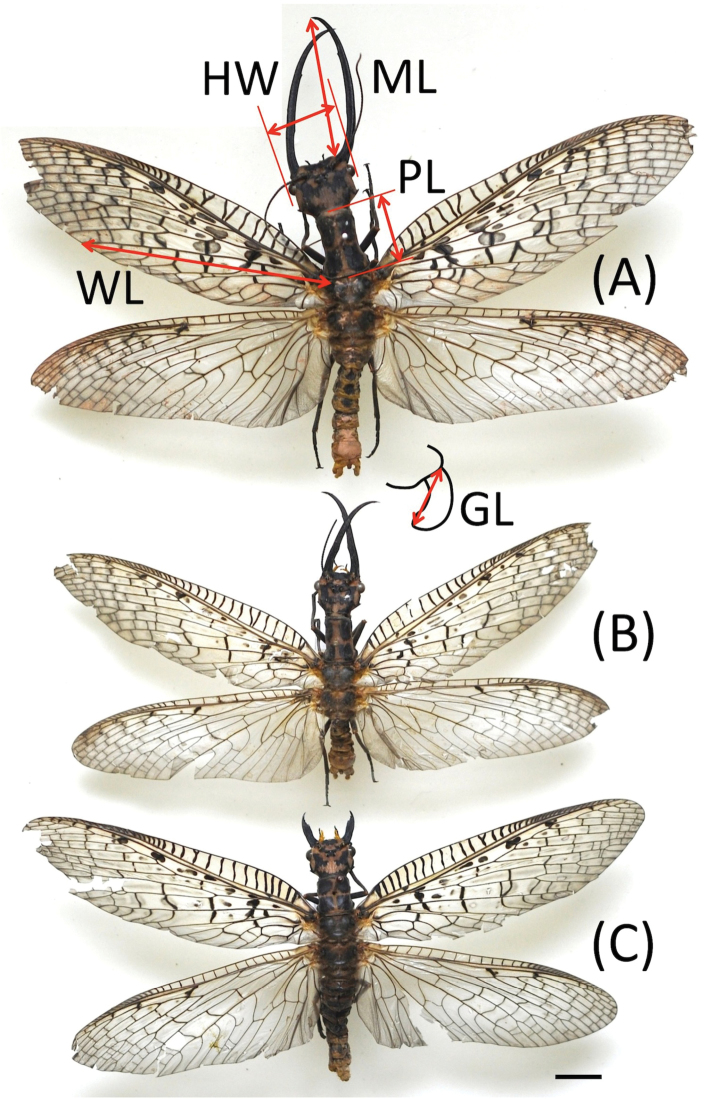
*Acanthacorydalisasiatica* in the dorsal view. **A** Large male **B** small male **C** female. Abbreviations: HW, head width; GL, genital (ectoproct) length in the lateral view; ML, mandible length; PL, prothorax length; WL, wing length. Scale bar: 10 mm.

Adult *A.asiatica* were obtained by rearing large larvae collected from Panzhihua, Sichuan Province, China. These larvae were collected on 12 April 2015 and brought to the laboratory to be kept in large plastic tanks (40 cm wide, 60 cm long, and 20 cm high) in which water obtained from underground was circulated 10 cm in depth. Chironomid larvae and shrimps were made available as food. Fully-grown larvae were replaced to the same-sized tanks but filled with wet soil (5 cm deep) for pupation. These tanks were covered with nets to prevent larvae escaping. When adults emerged, they were kept in a large rearing cage covered with fine wire nets (8 m wide, 25 m long, and 3.5 m high) in which several trees and grasses were planted and an artificial pond was set. Fruit (broken water melon) was given as food because the adult megalopterans can be reared by giving sugar solution, fermented milk, and/or fruit (Hayashi 1993, 1996, 1999; Villagomez and Contreras-Ramos 2017). In this cage, adults mated freely and laid egg masses. Water temperature in the larval tanks ranged from 10 to 20 °C, but air temperature and photoperiod were under natural conditions at Leshan, Sichuan, China. After the adults died, they were preserved as pinned dry specimens with wings spread (Fig. 1). The prothorax length (PL), head width between the outer left and right eye margins (HW), mandible length (ML), forewing length (WL) from the basal part to the tip of cubital vein, and ectoproct length of male genitalia (GL) were measured carefully with a digital slide caliper to the nearest 0.01 mm (Fig. 1). These five body parts are relatively hard structures, which allowed us to measure them even in the dried specimens. Usually, the right mandible, wing, and ectoproct were measured, but if the right ones were broken, the left ones were used for measurements, although in a few cases both were broken.

The mean value (± SD) and CV (%) were calculated for all measured parameters. As in Liu et al. (2015), PL was used as an index of the body size, and the allometric relationships with PL were calculated for HW, ML, WL, and GL after all data were log_10_-transformed. The regression slope was calculated by major axis regression for males and females, respectively, because the standard least-squares method tends to produce underestimations (McArdle 1988). The 95% and 99% confidence limits of the slope were also calculated. Sexual differences of the slope and intercept of regressions were tested by the analysis of covariance (ANCOVA) using likelihood ratio statistics for slopes and using Wald statistics for intercepts when the slopes are common (R Development Core Team 2017).

## Results

Males were larger than females on the average and there was a great size variation in male morphological traits; CV was greater in males than females (Table 1, Fig. 1). Among male traits, CV was largest in ML and smallest in GL, suggesting the mandible length varies individually, but the ectoproct length is rather stable.

**Table 1.** The prothorax length (PL), head width (HW), mandible length (ML), wing length (WL), and genital length (GL) of male and female *Acanthacorydalisasiatica*.

Sex	Males					Females			
Body part	PL (mm)	HW (mm)	ML (mm)	WL (mm)	GL (mm)	PL (mm)	HW (mm)	ML (mm)	WL (mm)
N	31	31	29	30	28	33	33	33	33
Mean	10.56	11.44	19.27	43.43	3.38	8.37	10.53	7.02	45.36
SD	1.86	1.70	5.93	4.51	0.33	0.87	0.85	0.82	4.19
CV%	17.63	14.83	30.76	10.38	9.73	10.37	8.07	11.74	9.23

**Figure 2.**
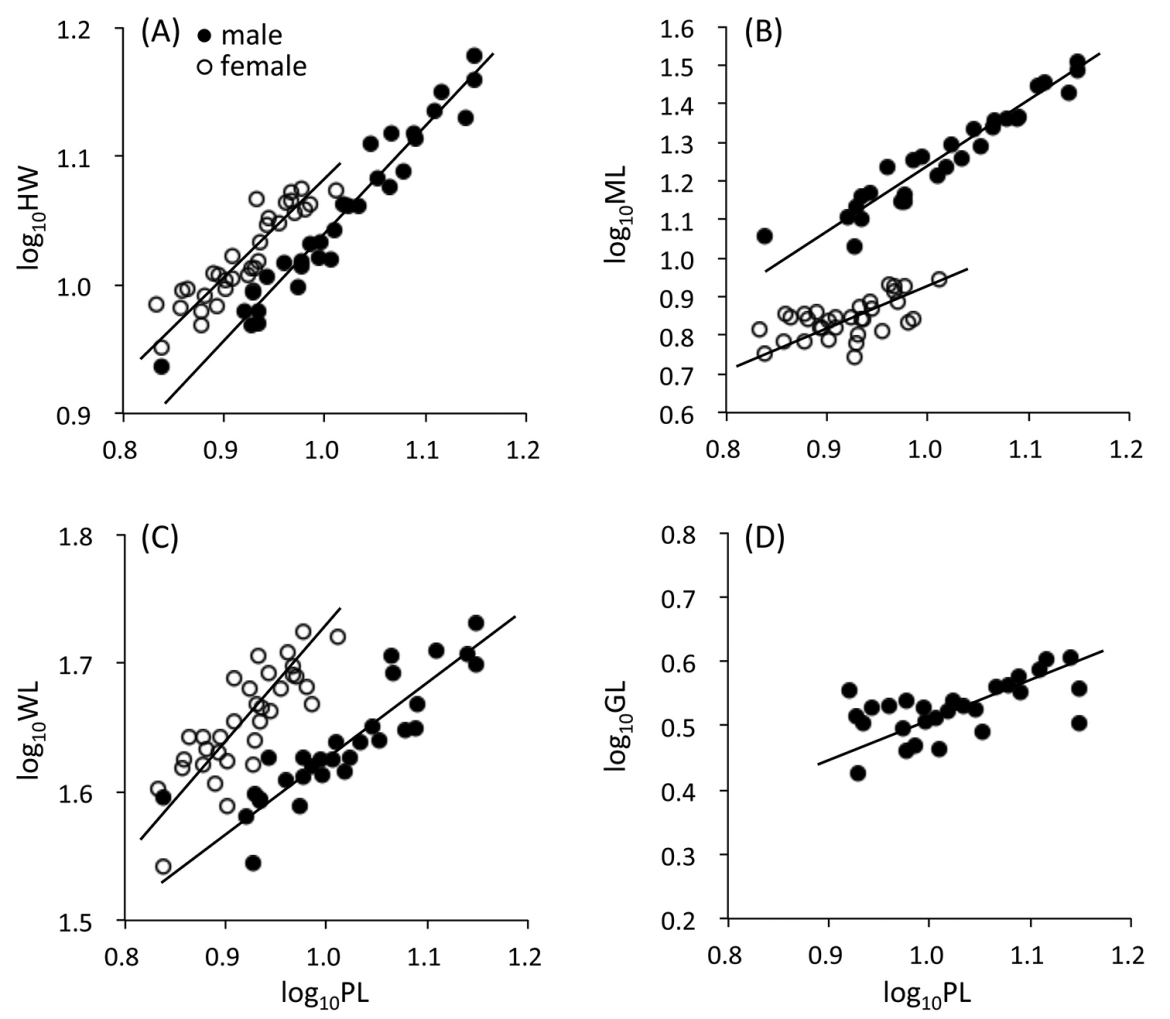
Log-log relationships between the prothorax length (PL) and the head width (HW in **A**), mandible length (ML in **B**), wing length (WL in **C**), and genital (ectoproct) length (GL in **D**) in male and female *Acanthacorydalisasiatica*. Regression lines: **A***y* = 0.829 *x* + 0.211 in males and *y* = 0.773 *x* + 0.310 in females **B***y* = 1.663 *x* − 0.428 in males and *y* = 1.113 *x* − 0.181 in females **C***y* = 0.582 *x* + 1.046 in males and *y* = 0.903 *x* + 0.823 in females **D***y* = 0.605 *x* − 0.094 in males. For statistical tests, see the text.

HW was correlated linearly with PL in log-log relationship both in males (*R*^2^ = 0.946, *P* < 0.0001) and females (*R*^2^ = 0.813, *P* < 0.0001), and the slope of the regression line was 0.829 in males and 0.773 in females (Fig. 2A). The slopes were less than 1 both in males and females (*P*s < 0.01), and did not differ between the sexes (ANCOVA, *P* = 0.434). However, the intercepts of the regressions differed between the sexes (*P* < 0.0001), suggesting that the head width is larger in females than males compared with same body size.

ML was always greater in males than females (Fig. 2B). ML was correlated linearly with PL in log-log relationship both in males (*R*^2^ = 0.916, *P* < 0.0001) and females (*R*^2^ = 0.342, *P* < 0.001), and the slope of the regression line was 1.663, significantly larger than 1 (*P* < 0.01), in males and 1.113, not different from 1 (*P* > 0.05), in females (Fig. 2B). The slopes differed significantly between the sexes (ANCOVA, *P* < 0.05), suggesting that the mandible length shows the positive allometry with their body size in males, but isometric in females.

WL was correlated linearly with PL in log-log relationship both in males (*R*^2^ = 0.774, P < 0.0001) and females (*R*^2^ = 0.664, *P* < 0.0001), and the slope of the regression line was 0.582, significantly lower than1 (*P* < 0.01) in males and 0.903, not different from 1 (*P* > 0.05), in females (Fig. 2C). The slopes differed significantly between the sexes (ANCOVA, *P* < 0.005), suggesting that the wing length shows the negative allometry with their body size in males, but isometric in females, and the wing is usually shorter in males than females compared with same body size.

GL was correlated linearly with PL in log-log relationship in males (*R*^2^ = 0.336, *P* < 0.005), and the slope of the regression line was 0.605 which was significantly lower than1 (*P* < 0.01), suggesting the ectoproct length shows the negative allometry with male body size (Fig. 2D).

## Discussion

Males of *Acanthacorydalis* species combat each other for access to females or limited resources (tree sap) that attract females (Liu et al. 2015). They use mandibles as weapons for their combat and the exaggerated mandibles may be favored by sexual selection, because males had longer mandibles than females and the allometry between body and mandible sizes was positive in males but isomeric in females. CV of the mandible length was also greatest among male traits examined in this study. Thus, mandibles are condition-dependent traits in males (House et al. 2015). In Megaloptera, positive allometry between body and weapon sizes is also reported in males of seven species of *Corydalus* in the Americas, three species of *Acanthacorydalis* in Asia, and two species of *Platyneuromus* in Central America (Liu et al. 2015; Álvarez et al. 2017; Ramírez-Ponce et al. 2017). In addition, 22 species of *Corydalus* in America are suggested to have elongated male mandibles fitting this allometric phenomenon (Contreras-Ramos 1998). In *Corydalus* and *Acanthacorydalis*, males have elongated mandibles, but in *Platyneuromus*, males have large flanges at the lateral sides of the head, spread like a fan, although the function is still unknown (Glorioso and Flint 1984; Ramírez-Ponce et al. 2017). The molecular phylogenetic tree of all genera of the subfamily Corydalinae of Megaloptera suggests that these three genera are included in the same lineage with the New World *Chloronia* which lacks any weapons in males, and therefore male weapons are thought to have evolved independently (Liu et al. 2015). In *Corydalusbidenticulatus*, positive allometry is reported between male body size and antenna length (Álvarez et al. 2017). In the present study, we cannot examine this, because most specimens were preserved after spending life in a large rearing cage and lost antennae.

For insects, resources used for adult body development are limited to those acquired during larval periods. Males suffer in how they allocate the limited resources to weapons and other body parts of adults. Much allocation to weapons is costly and, hence, trade-offs occur between weapons and other male traits such as wing size (e.g., Kawano 1995, 1997), ejaculate size (review by Simmons et al. 2017), nuptial gift size (Liu et al. 2015), and immune system (Körner et al. 2017; also see Pomfret and Knell 2006; McCullough and Emlen 2013). If males have two or more types of weapons, a trade-off is also important in allocating more resources to which type of weapons (Kojima and Lin 2017). In the present study, mandibles had positive but wings had negative allometries with body size in males, while both traits were isometric in females. Liu et al. (2015) reported the same tendency in three species of *Acanthacorydalis*, but no such tendencies in six species of *Corydalus* with enlarged male mandibles and two species of *Platyneuromus* with enlarged male head flanges, although specimens from multiple collecting sites were combined for analysis. Thus, in *Acanthacorydalis*, large males possess disproportionally large mandibles but small wings compared with small males, which is quite similar to the common results of stag beetles (Kawano 1997). However, such a trade-off must be demonstrated directly. Despite the accumulating evidence for resource allocation trade-offs in insects, these trade-offs are not universal (Simmons and Emlen 2006; McCullough and Emlen 2013). In the future, flight ability will be compared between large and small males to reveal the cost of disproportionally developed wings in *Acanthacorydalis*, compared with *Corydalus* and *Platyneuromus* likely to have proportionate wings.

Male genital size of *Acanthacorydalisasiatica* was only slightly influenced by variation in body size as supported by its lowest CV, and the allometric relationship was negative between body size and genital size. Although based on small sample size, the negative allometry was also obtained in American *Corydalusbidenticulatus* (Álvarez et al. 2017). The low influence of body size on genital size may be interpreted as evidence of stabilizing selection for it. Eberhard et al. (1998) considered that this is achieved by cryptic sexual selection and called it the one-size-fits-all hypothesis of insect male genitalia. If intermediate-sized genitalia provide a better fit or better tactile stimulation for more females in the population than small genitalia or large genitalia, the males with that-sized genitalia would leave more offspring, regardless of their body size (Eberhard et al. 1998, 2018). In insects, sperm are transferred to the female by direct ejaculation into the bursa copulatrix, deposition of a small spermatophore in the bursa copulatrix, or an external spermatophore attached to the female genital opening from which sperm enter the bursa copulatrix (Simmons 2001; Chapman 2013). Males of Megaloptera use the external spermatophore which is attached to the female within a few minutes at mating (Hayashi 1992, 1993, 1996, 1998; Liu et al. 2015). They lack the intromittent organ such an aedeagus (Liu et al. 2016). All the previous studies on genital allometries were done for insects with direct ejaculation or internally deposited spermatophores (reviewed by Eberhard et al. 1998, 2018; Eberhard 2009; Voje 2016). Thus, the present study reveals that the one-size-fits-all hypothesis also applies to male genitalia of Megaloptera with insemination via an externally attached spermatophore. Ectoprocts, which grasp the female abdominal tip, may be unnecessary to be enlarged with male body size. Insemination using an external spermatophore also occurs in crickets and bushcrickets in Orthoptera and some species in these taxa have male weaponry just like Megaloptera (e.g., Kelly 2005; Kim et al. 2011). It would be interesting to examine whether or not the same allometric relationship of genitalia occur between these sexually dimorphic Megaloptera and Orthoptera, which are distant phylogenetically (holometabolous and hemimetabolous, respectively), but similar in mating behavior.
